# *MALAT1* functions as a transcriptional promoter of *MALAT1::GLI1* fusion for truncated GLI1 protein expression in cancer

**DOI:** 10.1186/s12885-023-10867-6

**Published:** 2023-05-10

**Authors:** Taiji Hamada, Michiyo Higashi, Seiya Yokoyama, Toshiaki Akahane, Masanori Hisaoka, Hirotsugu Noguchi, Tatsuhiko Furukawa, Akihide Tanimoto

**Affiliations:** 1 Department of Pathology, Kagoshima University Graduate School of Medical and Dental Sciences, 8-35-1 Sakuragaoka, Kagoshima, 890-8544 Japan; 2 Department of Surgical Pathology, Kagoshima University Hospital, 8-35-1 Sakuragaoka, Kagoshima, 890-8544 Japan; 3 Center for Human Genome and Gene Analysis, Kagoshima University Hospital, 8-35-1 Sakuragaoka, Kagoshima, 890-8544 Japan; 4 Department of Pathology and Oncology, University of Occupational and Environmental Health, 1-1 Iseigaoka, Yahatanishi, Kitakyushu, 807-8556 Japan; 5 Center for the Research of Advanced Diagnosis and Therapy of Cancer, Kagoshima University Graduate School of Medical and Dental Sciences, 8-35-1 Sakuragaoka, Kagoshima, 890-8544 Japan

**Keywords:** *MALAT1*, *GLI1*, *MALAT1::GLI1* fusion gene, Plexiform fibromyxoma, 5' RACE, Luciferase assay, SUFU, Nuclear localization

## Abstract

**Background:**

The long non-coding RNA metastasis-associated lung adenocarcinoma transcript 1 (*MALAT1*) is a cancer biomarker. Furthermore, fusion of the *MALAT1* gene with glioma-associated oncogene 1 (*GLI1*) is a diagnostic marker of plexiform fibromyxoma and gastroblastoma; however, the function of this fusion gene remains unexplored.

**Method:**

In this study, we elucidate the structure and function of the *MALAT1::GLI1* fusion gene. To this end, we determined a transcriptional start site (TSS) and promoter region for truncated GLI1 expression using rapid amplification of the 5' cDNA end and a luciferase reporter assay in cultured cells transfected with a plasmid harboring the *MALAT1::GLI1* fusion gene.

**Results:**

We found that the TATA box, ETS1 motif, and TSS were located in *MALAT1* and that *MALAT1* exhibited transcriptional activity and induced expression of *GLI1* from the *MALAT1::GLI1* fusion gene. Truncated GLI1, lacking SUMOylation and SUFU binding sites and located in the nucleus, upregulated mRNA expression of GLI1 target genes in the hedgehog signaling pathway.

**Conclusions:**

We demonstrate a distinct and alternative function of *MALAT1* as a transcriptional promoter for expression of the *MALAT1::GLI1* fusion gene. Our findings will aid future research on *MALAT1* and its fusion gene partners.

**Supplementary Information:**

The online version contains supplementary material available at 10.1186/s12885-023-10867-6.

## Introduction

Although ~ 80% of human genes are transcribed into RNAs, less than 2% of RNAs are translated into peptides [1, 2]. Non-coding RNAs (ncRNAs), which are classified into housekeeping RNAs, such as transfer RNAs, ribosomal RNAs, small nuclear RNAs, and small nucleolar RNAs, and into microRNAs, PIWI-interacting RNAs, small interfering RNAs, and long non-coding RNAs (lncRNAs; > 200 nucleotides in length [2]) were once considered transcriptional noises or byproducts with no biological functions [3]. However, recent studies have demonstrated that ncRNAs play critical roles in various cellular functions, such as cell proliferation, differentiation, and apoptosis [4]. Dysregulation of ncRNAs is closely associated with increased cell proliferation, migration, and invasion in many cancer types, such as breast, lung, gastric, colorectal, and ovarian cancers [5, 6]. In addition, disordered ncRNAs can inhibit apoptosis, regulate epithelial-mesenchymal transition, and promote acquisition of drug resistance [6, 7]. Therefore, understanding ncRNA functions provides vital information for the development of ncRNA-based molecularly targeted therapy [7].

Metastasis-associated lung adenocarcinoma transcript 1 (*MALAT1*), which codes an 8.5-kb lncRNA located at chromosome 11q13.1, is a widely studied lncRNA that was first recognized in human non-small cell lung carcinoma [8]. *MALAT1*lncRNA regulates several signaling transduction pathways such as the MAPK/ERK, PI3K/Akt, and Wnt/β-catenin pathways and therefore modulates cancer cell proliferation, migration, invasion, and apoptosis [9, 10]. Furthermore, it promotes drug resistance by regulating the gene expression of *MDR*in glioblastoma against temozolomide, HIF-2α in hepatocellular carcinoma against 5-fluorouracil, adriamycin, and mitomycin C, and thymidine kinase in colorectal cancer against 5-fluorouracil [9]. Consequently, upregulated expression of *MALAT1*lncRNA is significantly related to poor prognosis for various cancer types [9–11]. Furthermore, *MALAT1*lncRNA is a useful diagnostic biomarker for various cancers because its increased expression is detectable in the exome, serum, and plasma fraction of peripheral blood [9].

Unlike *MALAT1* lncRNA expression, the fusion gene consisting of *MALAT1* and glioma-associated oncogene 1 (*GLI1*; located at chromosome 12q13.3) is a diagnostic molecular marker for the rare gastric tumor plexiform fibromyxoma (PFM) and gastroblastoma [12, 13]. GLI1, a transcriptional factor, is a key molecule in the hedgehog (HH) signaling pathway, and the dysregulated HH pathway is involved in the development and progression of many cancers, such as breast, pancreatic, and prostatic cancers [14, 15]. Following our previous report describing a case of esophageal PFM harboring the *MALAT1::GLI1*fusion gene coding an N-terminal truncated GLI1 protein (NtGLI1) [16], we hypothesize that *MALAT1* plays a pivotal role in the transcriptional regulation of truncated GLI1 expression.

The specific aim of this study was to clarify the structure and function of the *MALAT1::GLI1* fusion gene. To this end, we determined a potential transcriptional start site (TSS) and promoter region for truncated GLI1 expression using rapid amplification of the 5' cDNA end (5' RACE) and a luciferase reporter assay in cultured cells transfected with a plasmid harboring the *MALAT1::GLI1* fusion gene. We then demonstrated that the *MALAT1* region containing the TSS, TATA box, and ETS1 motif operates as a transcriptionally active promoter for the expression of truncated GLI1, indicating an alternative function of the *MALAT1* lncRNA-encoding gene. This study lays the foundation for further functional studies on *MALAT1* and its fusion gene partners.

## Materials and methods

### Cell culture and transfection

HEK293T cells were obtained from the JCRB Cell Bank (Osaka, Japan) and maintained in Dulbecco’s modified Eagle’s medium (FUJIFILM Wako Pure Chemical Corporation, Osaka, Japan) supplemented with 2 mM glutamine, 100 U/mL penicillin, 100 μg/mL streptomycin, and 10% fetal bovine serum (Sigma-Aldrich, St Louis, MO, USA) at 37 °C with 95% air and 5% CO_2_.

### Plasmid construction

The pcDNA3.1-His-hGLI1 plasmid was obtained from the RIKEN BioResource Research Center (RIKEN BRC, Ibaraki, Japan). *MALAT1* fragments (-1,307 to + 59, -1,307 to -56, and -1,307 to + 168) were cloned from the genomic DNA of HEK293T cells. *GLI1* cDNA (Nt*GLI1*, c.1_579del) was amplified from the pcDNA3.1-His-hGLI1 plasmid. These fragments were assembled using NEBuilder HiFi DNA Assembly Master Mix (New England Biolabs, Ipswich, MA, USA) to generate a *MALAT1::GLI1* fusion gene. *GLI1* **Δ**N cDNA (c.1_384del) was excised from the pcDNA3.1-His-hGLI1 plasmid, and the p.K180R mutation was introduced by PCR. For the Myc-tagged GLI1 expression plasmid, full-length GLI1 and Nt*GLI1* cDNAs were inserted into the BamHI site of the pCMV-3Tag-2c vector (Agilent Technologies, Santa Clara, CA, USA). The FLAG-tagged SUFU expression plasmid was generated from *SUFU* cDNA (RIKEN BRC), which was inserted into the EcoRI-BglII sites of the p3xFLAG-CMV-10 vector (Sigma-Aldrich).pGL4-phGLI1 was obtained from RIKEN BRC for the luciferase reporter assay. A *MALAT1* part of the fusion gene (t*MALAT1*) fragment (-1,307 to + 59) was inserted into the pGL4.10 vector (Promega, Madison, WI, USA) immediately upstream of the luciferase gene, using NEBuilder HiFi DNA Assembly Master Mix (New England Biolabs). The 5'-serial deletion mutations of t*MALAT1* were synthesized as shown in Supplementary Table 2 (Integrated DNA Technologies, Coralville, IA, USA) and inserted into pGL4.10. Transcription factor-binding motifs in t*MALAT1* were analyzed using the JASPAR database (https://jaspar.genereg.net), and pGL4 plasmids containing mutations in the motifs were generated using PCR-based mutagenesis. *MALAT1p* was cloned using PCR and inserted into the 5' site of t*MALAT1* in the pGL4 reporter. All primers used for plasmid construction are listed in Supplementary Table 3.

### Luciferase reporter assay

Cells were seeded at a density of 1 × 10^4^ cells/well in 96-well plates. The following day, using Lipofectamine 3000 (Thermo Fisher Scientific, Waltham, MA, USA), the cells were transfected with 100 ng of the reporter and 5 ng of the Renilla luciferase control vector (pRL-TK, TOYO B-Net, Tokyo, Japan). For SUFU and GLI1 expression assays, the cells were transfected with 50 ng of SUFU and/or GLI1 expression plasmids. On the following day, the cells were lysed and luciferase activity was measured using the Dual-Luciferase Reporter Assay System (Promega). Luciferase activity was quantified using a PHELIOS luminometer (ATTO, Tokyo, Japan).

### Reverse transcription-quantitative PCR (RT-qPCR) analysis

Cells were seeded at a density of 5 × 10^4^ cells/well in 24-well plates. The following day, using Lipofectamine 3000, the cells were transfected with 500 ng of the GLI1 expression plasmid. After three days, total RNA was extracted using the RNeasy Plus kit (QIAGEN, Hilden, Germany) and reverse-transcribed to cDNA using the ReverTra Ace qPCR RT Kit & Master Mix (TOYOBO). RT-qPCR was conducted using KOD SYBR qPCR Mix (TOYOBO) on the LightCycler 480 system (Roche Diagnostics, Basel, Switzerland). The primers used in this study are listed in Supplementary Table 4. Gene expression was analyzed using the comparative Ct method with hypoxanthine–guanine phosphoribosyl transferase gene expression used as a reference.

### Rapid amplification of the 5'cDNA end

Total RNA from cells transfected with the *MALAT1::GLI1* gene plasmid was extracted using the same procedure as that used for the RT-qPCR analysis of mRNA expression. Briefly, 5' RACE was performed using the *GLI1*-specific primers listed in Supplementary Table 5 and a 5' RACE kit (SMARTer RACE 5'/3' Kit; Takara Bio Inc., Shiga, Japan).

### Western blotting and immunoprecipitation

Cells were seeded at a density of 5 × 10^5^ cells/well in 6-well plates. The following day, using Lipofectamine 3000, the cells were transfected with 2.5 μg of the GLI1 expression plasmid. After three days, the cells were precipitated with 10% trichloroacetic acid on ice for 30 min. The precipitates were then washed with cold phosphate-buffered saline (PBS) and dissolved in lysis buffer (7 M urea, 2 M thiourea, 3% CHAPS, and 1% Triton X-100). The lysates were loaded onto polyacrylamide gels, and the separated proteins were transferred to polyvinylidene difluoride membranes. The membranes were blocked using 5% nonfat dried milk in Tris-buffered saline with 0.1% Tween 20 for 1 h and then incubated overnight at 4 °C with a primary antibody diluted in Can Get Signal solution 1 (TOYOBO) and either horseradish peroxidase-conjugated goat anti-guinea pig, goat anti-rabbit, or mouse secondary antibodies (Cell Signaling Technology [CST], Danvers, MA, USA) for 1 h at room temperature. The antibodies used in this study (all from CST, except for anti-FLAG) were anti-GLI1 (#3538), anti-Myc-tag (#2278), anti-SUFU (#2522), anti-β-actin (#4970), anti-MEK1 (#4694), anti-histone deacetylase 1 (#2062), and anti-FLAG (F1804, Sigma-Aldrich). The anti-human GLI1 antibody recognizes the amino acid residues surrounding Gly420. Proteins were detected with SuperSignal West Pico PLUS Chemiluminescent Substrate (Thermo Fisher Scientific) or Clarity Max Western ECL Substrate (Bio-Rad, Hercules, CA, USA). Densitometry analysis was performed using the Ez-Capture MG and CS Analyzer 3.0 software (ATTO).

### Immunoprecipitation

Cells were seeded at a density of 1 × 10^6^ cells in 60-mm dishes. The following day, using Lipofectamine 3000, the cells were transfected with 3 μg of Myc-GLI1 and FLAG-SUFU expression plasmids. After three days, the cells were lysed using IP Lysis Buffer (Thermo Fisher Scientific), and the lysates were incubated overnight at 4 °C with rabbit anti-Myc-tag and mouse anti-FLAG antibodies. The immunocomplex was captured using protein A or G beads (Tamagawa Seiki Co. Ltd., Nagano, Japan), and the eluted samples were subjected to western blotting.

### Immunofluorescence study

Cells were seeded at a density of 1 × 10^5^ cells/well in a four-well chamber slide (Millicell EZ SLIDES, Merck Millipore, Burlington, MA, USA). The following day, using Lipofectamine 3000, the cells were transfected with 250 ng of GLI1 and SUFU expression plasmids. After two days, the cells were fixed with 4% paraformaldehyde for 15 min at room temperature, rinsed with PBS, and incubated in a blocking solution (3% bovine serum albumin [BSA] and 0.3% Triton X-100 in PBS) for 60 min at room temperature. Slides were incubated with primary antibodies (anti-Myc and anti-FLAG antibodies) in a dilution buffer (1% BSA and 0.3% Triton X-100 in PBS) at 4 °C. After washing with PBS, the slides were incubated with anti-mouse IgG (Alexa Fluor 488 conjugate, #4408, CST) and anti-rabbit IgG (Alexa Fluor 647 conjugate, #4414, CST) for 1–2 h at room temperature. After washing with PBS, the coverslips were mounted with Fluoro-KEEPER Antifade Reagent with DAPI (Nacalai Tesque, Kyoto, Japan). Images were obtained using a fluorescence microscope (Axio Observer, ZEISS, Oberkochen, Germany).

### Subcellular fractionation analysis

Cells were seeded at a density of 3 × 10^6^ cells in 100-mm dishes. The following day, using Lipofectamine 3000, the cells were transfected with 7.5 μg of Myc-GLI1 and 7.5 μg of FLAG-SUFU expression plasmids. After three days, the cell lysates were fractionated into cytoplasmic and nuclear fractions using a commercial kit (EzSubcell Fraction, ATTO). The samples were subsequently used for western blot analysis.

### Histology and immunohistochemistry for clinical cases

Formalin-fixed paraffin-embedded tissue sections were subjected to hematoxylin–eosin staining and immunohistochemistry for the detection of GLI1 expression using the anti-GLI1 antibody (#3538, CST) in esophageal [16] and gastric PFM cases.

### *In-silico* analysis

The ChimerDB 4.0 database (http://www.kobic.re.kr/chimerdb/) was used to detect possible *MALAT1*fusion genes and predict fusion gene structures [17].

### Statistical analysis

All experiments were performed 3–6 times independently; their results are presented as the mean ± standard error of the mean. Statistical significance was determined using the unpaired one-tailed Student's *t*-test. The results were considered significant at *p* < 0.05.

## Results and discussion

In this study, we demonstrate that the *MALAT1* lncRNA-encoding region shows transcriptional regulatory activity for the *MALAT1::GLI1* fusion gene. *MALAT1* contains a potential TATA box, ETS1 motif, and TSS as a transcriptional regulatory region for the expression of NtGLI1, indicating an alternative and distinct function of the *MALAT1* lncRNA-encoding gene. As for a limitation of the present study, we have to consider a possibility that a cryptic or aberrant splicing, which occurs in plasmid-based expression study using fusion genes harboring exon-exon junctions [18], undermine or modify the transcriptional cis-regulator function of t*MALAT1*.

### Structure and function of the *MALAT1::GLI1* fusion gene

**Fig. 1:**
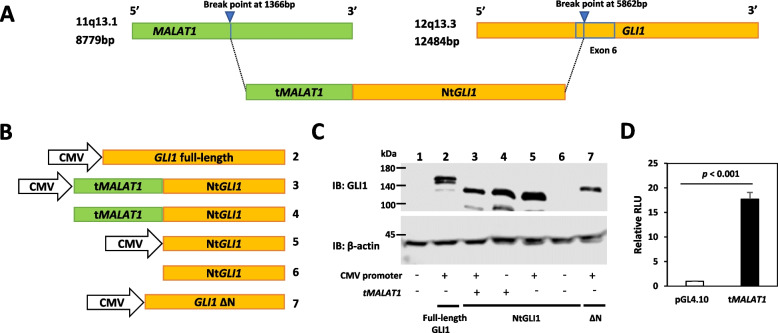
Structure and function of the *MALAT1::GLI1* fusion gene. **A** Presumed structure of the *MALAT1::GLI1* fusion gene cloned from an esophageal plexiform fibromyxoma. **B** Constructs of GLI1 expression plasmids containing the CMV promoter, *tMALAT1* region, both, or none. **C** Western blotting analysis of GLI1 expression. Lane numbers correspond to those in (**B**). Lysates from cells transfected with the promoter-free construct show no GLI1 expression (lane 6), whereas those from cells transfected with constructs harboring NtGLI1 and the CMV promoter (lane 5), the *tMALAT1* region (lane 4), or both (lane 3) show considerable NtGLI1 expression. The molecular size of NtGLI1 is smaller than that of full-length GLI1 (lane 2). GLI1 ΔN is expressed under the CMV promoter (lane 7). **D** The *tMALAT1* region exhibits basal transcriptional activity in the pGL4.10 luciferase reporter assay. *MALAT1*, metastasis-associated lung adenocarcinoma transcript 1; *GLI1*, glioma-associated oncogene 1; Nt*GLI1*, N-terminal truncated *GLI1*; CMV, cytomegalovirus

In our previous study, we revealed that the structure of the *MALAT1::GLI1* fusion gene exhibits a breakpoint with an overlapping sequence of GAGGAA (1,361–1,366 bp in *MALAT1* and 5,862–5,867 bp in *GLI1* exon 6; Fig. 1A). Given this overlapping sequence, we were unable to determine the exact breakpoint. Therefore, for convenience in this study, we considered that the breakpoint localizes at 1,366 bp in the *MALAT1* gene and 5,862 bp in *GLI1*exon 6. The fusion gene shows an in-frame amino acid reading from GLI1 Met199 for NtGLI1 [16]. Based on our speculation that the *MALAT1* part of the fusion gene (t*MALAT1*) may contain a TSS and upstream promoter region, we first performed expression analysis using various Nt*GLI1* constructs (Fig. 1B). Plasmids harboring the cytomegalovirus (CMV) promoter (construct 5), t*MALAT1* (construct 4), or both (construct 3) showed comparable expression levels of NtGLI1 (lanes 3–5) to the CMV promoter-driven full-length GLI1 plasmid (lane 2; Fig. 1C). In contrast, constructs with neither the CMV promoter nor t*MALAT1* showed no NtGLI1 expression (lane 6). The CMV promoter-driven full-length GLI1 construct had a larger GLI1 than NtGLI1 (lane 2). GLI1 **Δ**N, which lacks the N-terminal 128 amino acids [19], was expressed under the control of the CMV promoter (lane 7).

**Fig. 2:**
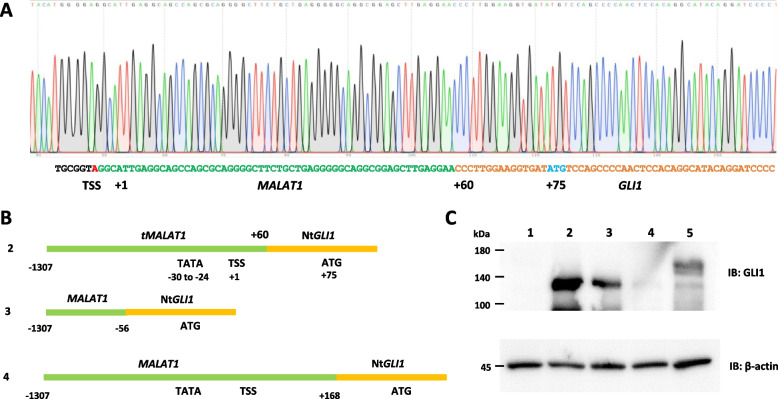
Structure and function relation of *tMALAT1* for NtGLI1 expression. **A** Sequence of the *MALAT1::GLI1* fusion gene. The transcriptional start site (TSS) is determined by 5'-RACE and numbered as + 1. Consequently, the fusion breakpoint is located at + 60, and the start codon is located at + 75. **B** Constructs of GLI1 expression plasmids with various length of the *MALAT1* region as a promoter. **C** Western blotting analysis of GLI1 expression. Lane numbers correspond to those in (**B**). Lysates from cells transfected with NtGLI1 expression plasmids with shorter (lane 3) or longer (lane 4) *MALAT1* sequences exhibit lower or no NtGLI1 expression, respectively. Cells transfected with constructs harboring the *tMALAT1* region show higher NtGLI1 expression (lane 2). Full-length GLI1 is expressed under the CMV promoter (lane 5). *MALAT1*, metastasis-associated lung adenocarcinoma transcript 1; *GLI1*, glioma-associated oncogene 1; Nt*GLI1*, N-terminal truncated *GLI1*; TSS, transcriptional start site; CMV, cytomegalovirus; 5'-RACE, rapid amplification of the 5' cDNA end

Next, we constructed a luciferase reporter plasmid containing t*MALAT1* in the pGL4.10 basic vector, in which the t*MALAT1*-luciferase construct exhibited considerable transcriptional activity (Fig. 1D). Analysis of 5' RACE using *MALAT1::GLI1* revealed that the TSS was located at 59 bp upstream from the breakpoint of the *MALAT1::GLI1* fusion gene (Fig. 2A). Furthermore, a consensus site for the TATA box was recognized at 30 bp upstream of the TSS, after reviewing previously determined sequences [16].

### Structure and function of t*MALAT1* for NtGLI1 expression

The gene structure of *MALAT1::GLI1* is schematically shown in Fig. 2A and as the no. 2 construct in Fig. 2B. The nucleotide number is renumbered as + 1 from the TSS, and the TATA box and ATG are subsequently located at -30 to -24 and + 75, respectively. Compared to the construct harboring the original *MALAT1::GLI1* (lane 2, Fig. 2C), the construct harboring the 3'-region deletion (construct 3, Fig. 2B) and the 3'-region elongation (construct 4, Fig. 2B) showed reduced NtGLI1 expression (lanes 3 and 4, Fig. 2C). The NtGLI1 expression from the construct 3 (construct 3, Fig. 2B and lane 3, Fig. 2C) indicated a presence of another potential TSS in the upstream region of the *MALAT1* promoter.

**Fig. 3:**
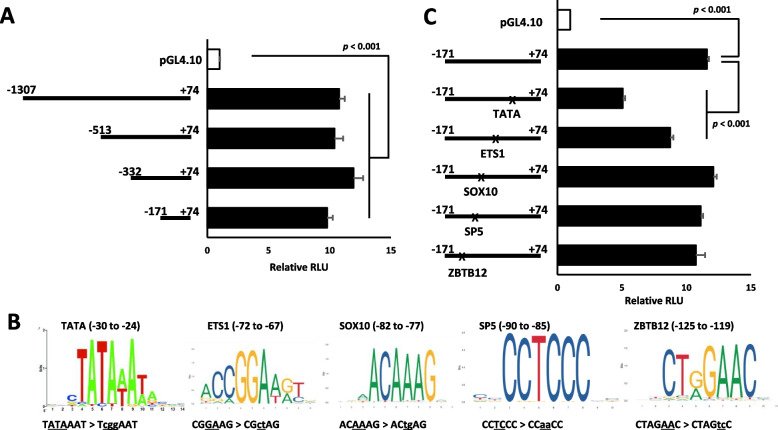
Deletion and mutation analyses for *tMALAT1* transcriptional activity. **A** Serial deletion studies for *tMALAT1* region promoter activity. Luciferase activities are not decreased in the serial deletion up to -171. **B** The -171 to + 74 region of the *MALAT1::GLI1* fusion gene contains consensus motifs of the TATA box, ETS1, SOX10, SP5, and ZBTB12. Mutations are introduced to the motifs by base substitution in the -171/ + 74 reporter construct. **C** Mutation analysis reveals that mutations in the TATA box and ETS1 reduce basal promoter activity from the *tMALAT1* sequence. *MALAT1*, metastasis-associated lung adenocarcinoma transcript 1; *GLI1*, glioma-associated oncogene 1

For further study of the t*MALAT1* sequence as a transcriptional promoter, luciferase reporter constructs containing -1,307 to + 74 of the *MALAT1::GLI1* fusion gene were used for deletion and mutation analyses. Basal promoter activity was not decreased by 5' serial deletions up to -171 (Fig. 3A). The -171/ + 74 construct included consensus sites of ETS1, SOX10, SP5, and ZBTB12 in the upstream proximal region of the TATA box. When these consensus sites were mutated by two or three nucleotide substitutions (Fig. 3B), the mutations introduced in the TATA box and ETS1, but not other mutations, reduced luciferase activity (Fig. 3C).

**Fig. 4:**
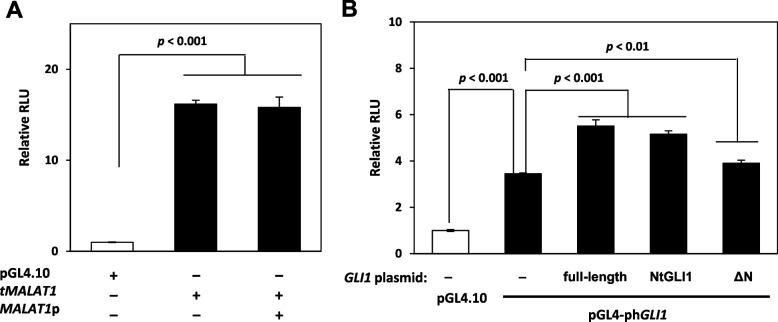
(**A**) Effect of *MALAT1p* on *tMALAT1* transcriptional activity. **A** The *MALAT1p* sequence is inserted at the upstream of *tMALAT1* in luciferase reporter constructs. Transcriptional activities are comparable, regardless of the presence and absence of upstream *MALAT1p*. (**B**) GLI1-mediated GLI1 transcription. **B** *GLI1* promoter luciferase assay performed by co-transfection of various GLI1 expression plasmids. Expression of full-length GLI1, NtGLI1, and GLI1 ΔN enhances the basal activity of the *GLI1* promoter luciferase reporter. *MALAT1*, metastasis-associated lung adenocarcinoma transcript 1; *GLI1*, glioma-associated oncogene 1; Nt*GLI1*, N-terminal truncated *GLI1*; *MALAT1p*, original MALAT1 promoter

*GLI1* gene translocation to the *MALAT1* gene locus generates the formation of *MALAT1::GLI1*fusion at chromosome 11q13.1 [12, 13]. Therefore, the possible genomic structure would be arranged in the following order from the 5'-proximal to the 3'-distal direction: original *MALAT1* promoter (*MALAT1p*), t*MALAT1*, and Nt*GLI1*. To investigate the effect of *MALAT1p* on t*MALAT1* transcriptional activity, luciferase reporter constructs containing *MALAT1p* placed upstream of the -1,307 to + 74 region of t*MALAT1*were generated and used for further studies [20]. The promoter activities of the t*MALAT1*-luciferase reporter constructs were comparable in the presence and absence of the upstream *MALAT1p* region (Fig. 4A).

*MALAT1*lncRNAs have various cellular functions that are mediated through post-transcriptional interaction with mRNAs, DNAs, and transcriptional regulators [9, 10]. However, the transcriptional regulatory activity of *MALAT1*after fusion gene formation has not been reported previously. Fusion gene formation between lncRNA-encoding and transcriptional factor-encoding genes is an unusual combination for gene rearrangement [21, 22]. Moreover, the observation that the lncRNA-encoding *MALAT1* region exhibits transcriptional regulatory activity after fusion with *GLI1* represents an exceptionally unique mode of gene regulation.

### GLI1 target gene expression and GLI1 functional domains

**Fig. 5:**
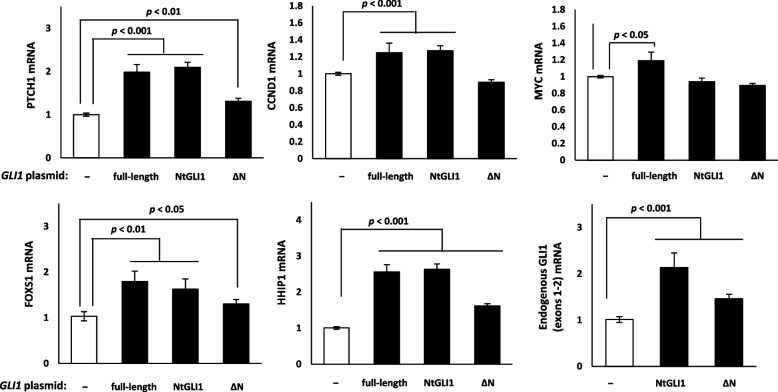
Induced expression of *PTCH1*, *FOXS1*, *CCND1*, *HHIP1*, *MYC*, and *GLI1*. Full-length GLI1 expression upregulates the mRNA expression of *PTCH1*, *FOXS1*, *CCND1*, *HHIP1*, and *MYC*. NtGLI1 expression upregulates the mRNA expression of all genes, except for *MYC*. GLI1 ΔN shows increased mRNA expression of all genes, except for *CCND1* and *MYC*. Endogenous *GLI1* mRNA expression is upregulated by both NtGLI1 and GLI1 ΔN. *MALAT1*, metastasis-associated lung adenocarcinoma transcript 1; *GLI1*, glioma-associated oncogene 1; Nt*GLI1*, N-terminal truncated *GLI1*

As GLI1 is a transcriptional factor that regulates the HH signaling pathway and upregulates the expression of *GLI1*, *PTCH1*, *FOXS1*, *CCND1*, *MYC*, and other target genes [23, 24], we studied the effects of NtGLI1 on the expression of these genes. In particular, NtGLI1 transactivates *GLI1* expression in a positive feedback manner. Therefore, we performed a luciferase assay using the GLI1 expression plasmid and *GLI1* promoter luciferase constructs. When the GLI1 expression plasmid was co-transfected with the luciferase reporter, over expression of full-length GLI1, NtGLI1, and GLI1 **Δ**N enhanced the basal activity of the *GLI1* promoter luciferase reporter gene (Fig. 4B). Full-length GLI1 upregulated the mRNA expression of *PTCH1*, *FOXS1*, *CCND1*, *HHIP1*, and *MYC*. Moreover, NtGLI1 upregulated the mRNA expression of these genes, except for *MYC*. GLI1 **Δ**N showed increased mRNA expression of *PTCH1*, *FOXS1*, and *HHIP1*, except for *CCND1* and *MYC*. Moreover, endogenous *GLI1* mRNA expression was upregulated by NtGLI1 and GLI1 **Δ**N (Fig. 5).

**Fig. 6:**
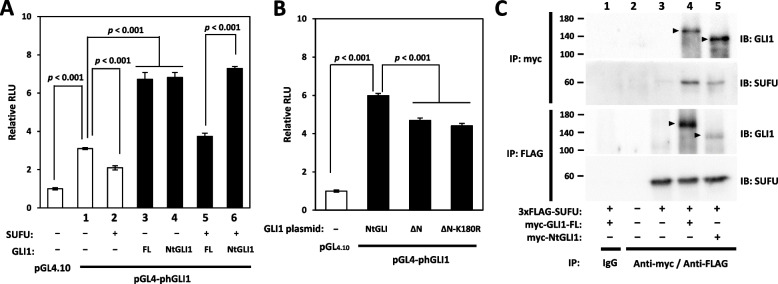
(**A**, **B**) Effects of SUFU-binding and SMOylation on *GLI1* transcription by GLI1 transactivation. **A** GLI1 transactivation for the *GLI1* promoter is decreased with SUFU co-expression (lanes 1 and 2). *GL11* promoter activity is comparable between the co-transfection of full-length GLI1 (FL) and NtGLI1 with no SUFU co-expression (lanes 3 and 4). However, *GLI1* promoter activity with SUFU co-expression is higher in cells expressing NtGLI1 than in those expressing full-length GLI1 (lanes 5 and 6). **B** GLI1 ΔN activity of transactivation in the *GLI1* promoter luciferase assay is the same, regardless of the presence or absence of Lys180Arg mutation. (**C**) Immunoprecipitation studies of GLI1 and SUFU. **C** In the upper panel, protein complexes are immunoprecipitated (IP) by anti-myc antibody against myc-GLI1-FL or myc-NtGLI1, while in the lower panel, protein complexes are immunoprecipitated by the anti-FLAG antibody against 3xFLAG-SUFU. GLI1 and SUFU proteins are detected (IB) using specific antibodies for each. Co-IP of GLI1-FL with SUFU are substantially detected (lane 4). In contrast, SUFU-binding domain-deficient NtGLI1 shows decreased co-IP with SUFU proteins (lane 5). *MALAT1*, metastasis-associated lung adenocarcinoma transcript 1; *GLI1*, glioma-associated oncogene 1; Nt*GLI1*, N-terminal truncated *GLI1*; SUFU, suppressor of fused homolog; FL, full length GLI1; IP, immunoprecipitation; IB, immunoblotting

NtGLI1 and full-length GLI1 activities for transactivation were comparable during *GLI1*transcription, indicating the presence of high endogenous wild-type GLI1 activity that may conceal NtGLI1 transactivation. The suppressor of fused homolog (SUFU) was overexpressed to sequester full-length GLI1 to reduce endogenous GLI1 activity, as NtGLI1 lacks the SUFU-binding domain located in amino acids 120–124 [25]. The *GLI1* promoter luciferase assay showed decreased basal transcriptional activity with SUFU co-expression (Fig. 6A). *GLI1* promoter activities were comparable between the co-transfection of full-length GLI1 and NtGLI1-expressing constructs with no SUFU co-expression. However, when SUFU was co-expressed, *GLI1* promoter activity was significantly higher in NtGLI1 co-transfected cells than in full-length GLI1 co-transfected cells.

In the *GLI1* promoter assay, NtGLI1 showed higher transactivation activity than GLI1 **Δ**N and GLI1 **Δ**N with the Lys180Arg mutation (Fig. 6B). SUMOylation of GLI2 and GLI3 at Lys180 enhances GLI1 transactivation by GLI1 liberation from SUFU [26]. GLI1 **Δ**N transactivation activity in the *GLI1* promoter luciferase assay was unaffected by the presence or absence of the Lys180Arg mutation that abolishes SUMOylation. Therefore, SUMOylation does not affect NtGLI1 or GLI1 **Δ**N activity, as NtGLI1 and GLI1 **Δ**N are deficient in the SUFU-binding domain.

### Co-immunoprecipitation analysis of GLI1 and SUFU

To understand the lack of binding of NtGLI1 to SUFU, we performed immunoprecipitation analysis using FLAG-tagged-SUFU and Myc-tagged-GLI1 expression constructs (Fig. 6C). Immunoprecipitation using anti-FLAG (for SUFU) and anti-Myc (for full-length GLI1 and NtGLI1) antibodies showed co-precipitation of SUFU with full-length GLI1, which was detected by specific anti-SUFU and anti-GLI1 antibodies (lane 4). When SUFU-binding domain-deficient NtGLI1 was co-transfected with the SUFU expression plasmid, the co-precipitation of SUFU and NtGLI1 proteins was reduced (lane 5).

### Subcellular localization of NtGLI1

**Fig. 7:**
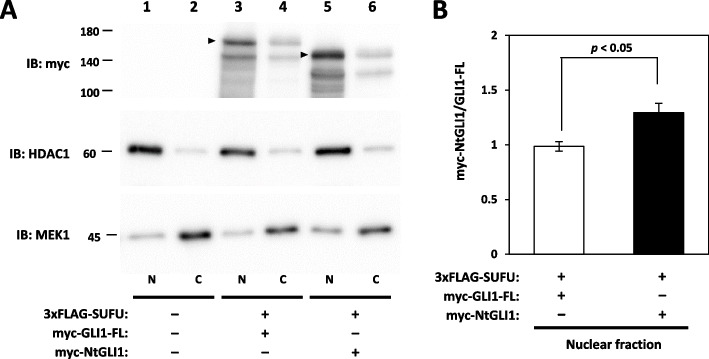
Subcellular localization of NtGLI1. **A** NtGLI1 (myc-NtGLI1) is predominantly located in the nuclear fraction (N) of cells co-transfected with the SUFU-expressing plasmid (lanes 5 and 6). Full-length GLI1 (myc-GLI1-FL) is also located in the nuclear fraction of cells co-transfected with the SUFU-expressing plasmid, but its amount is lower than that of NtGLI1 (lanes 3 and 4). **B** Densitometry of the nuclear fraction. The amount of the nuclear fraction of NtGLI1 is significantly higher than that of full-length GLI1. HDAC1 expression is detected as a reference for the nuclear fraction (N). MEK1 expression is detected as a reference for the cytoplasmic fraction (C). *GLI1*, glioma-associated oncogene 1; NtGLI1, N-terminal truncated GLI1; SUFU, suppressor of fused homolog; IB, immunoblotting

**Fig. 8:**
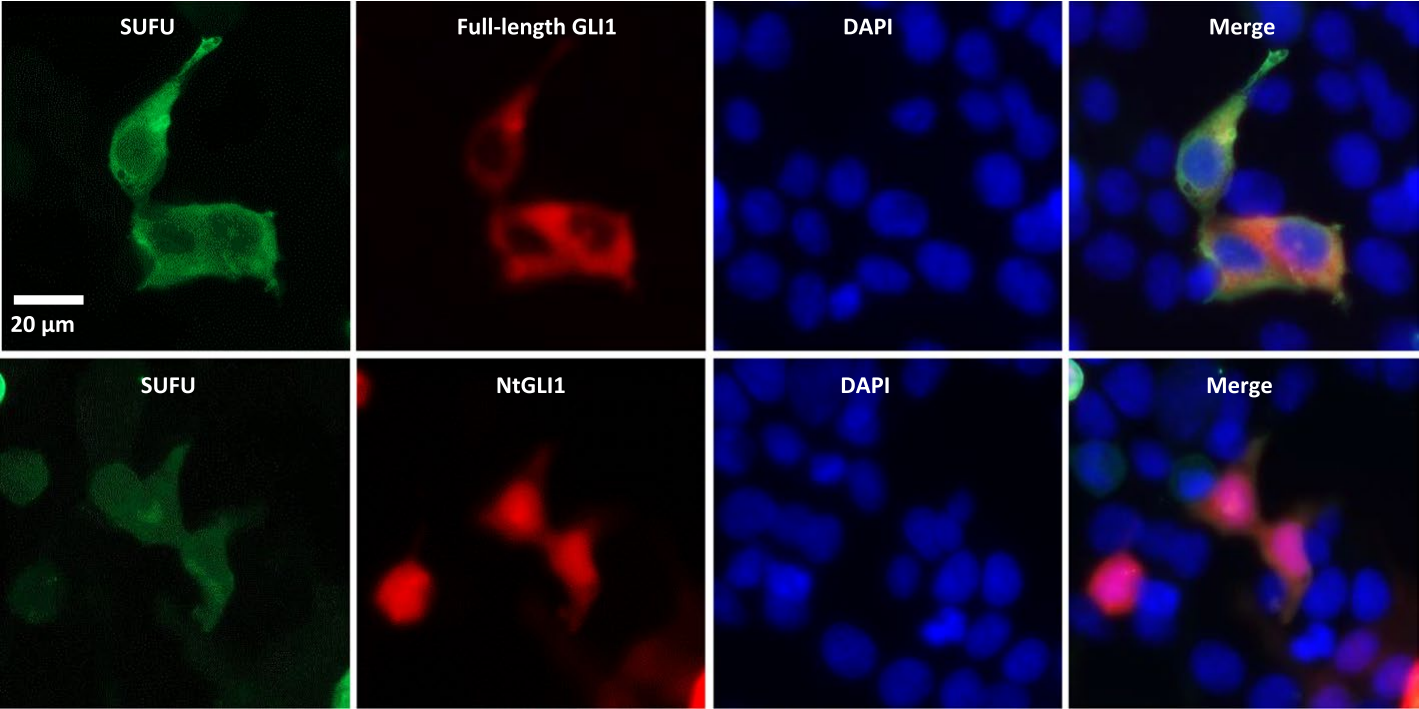
Immunofluorescence analysis of the subcellular localization of full-length GLI1 and NtGLI1. Myc-tagged full-length GLI1 or NtGLI1 are expressed and detected using an anti-Myc-tag antibody. NtGLI1, but not full-length GLI1, is mainly located in the nucleus. GLI1, glioma-associated oncogene 1; NtGLI1, N-terminal truncated GLI1

Subcellular localization was monitored by western blotting of subcellular fractions and immunofluorescence analysis in cells transfected with Myc-tagged full-length GLI1 and NtGLI1 constructs. Upon co-transfection of the SUFU-expressing plasmid, NtGLI1 was predominantly detected in the nuclear fraction (lanes 5 and 6; Fig. 7A). Full-length GLI1 was also located in the nuclear fraction (lanes 3 and 4; Fig. 7A); however, the amount of the nuclear fraction of NtGLI1 was significantly higher than that of the nuclear fraction of full-length GLI1 (Fig. 7B). Immunofluorescence analysis using an anti-Myc antibody demonstrated that NtGLI1, but not full-length GLI1, was mainly located in the nuclei (Fig. 8). In combination with the results of immunoprecipitation analysis, the N-terminal truncation and subsequent SUFU-binding domain deficiency, but not the SUMOylation site, may contribute to the acquisition of the active function of NtGLI1 mediated through enhanced nuclear translocation.

### *MALAT1::GLI1* fusion as a diagnostic marker for PFM and gastroblastoma

Gastric PFM and gastroblastoma harbor *MALAT1::GLI1*fusions with the same open reading frame starting from Met199 [12, 13, 16]. Thus, these fusion genes are speculated to encode the same N-terminal truncated GLI1, despite their different breakpoints. These fusion genes consist of a longer *MALAT1* region with an extra-sequence at the 3'-end as compared to the *MALAT1::GLI1* fusion gene presented in this study. As both shortening and elongation of the 3'-*MALAT1* sequence of the *MALAT1::GLI1* fusion gene decreased or abolished NtGLI1 protein expression (Fig. 2C), the presence of the TATA box, ETS1 motif, and TSS and its location at an optimal distance from the start codon might be necessary for the maximal transcriptional regulatory activity of NtGLI1. However, considering that immunohistochemical analysis showed nuclear overexpression of GLI1, the transcriptional activity of the truncated NtGLI1 protein would likely be maintained in tumor cells, in which the *MALAT1::GLI1* fusion gene harbors a longer *MALAT1*3'-region [12, 13]. Nevertheless, the relationship between the fusion gene structure and its transcriptional regulatory functions remains controversial. However, although the mechanism(s) of transcriptional regulation by the *MALAT1* region should be further clarified, *MALAT1::GLI1*fusion gene detection should prove useful as a molecular marker of PFM and gastroblastoma, showing a higher sensitivity than GLI1 immunohistochemistry [13, 27]. Our example of PFM [16] showed extensive nuclear expression of GLI1 in the tumor cells (Supplementary Fig. 1). As *PTCH1::GLI2*fusion is another diagnostic molecular marker for gastroblastoma [28, 29], dysregulation of the HH signaling pathway may be a common molecular marker for PFM and gastroblastoma.

### *GLI1* fusion genes as a diagnostic marker for other tumors

A few studies reported the *GLI1* fusions with protein-coding regions of 5’ fusion partner genes, such as *PTCH1* and *ACTB*, in rare glomoid neoplasms of the soft tissue [30, 31]. However, a combined approach of protein expression study, mRNA sequencing, transcriptional assay, and 5'-RACE, would be required to clarify whether this type of fusion genes express the truncated GLI1 or chimeric proteins.

### *MALAT1* fusion genes as diagnostic markers for other tumors

Various fusion gene partners for *MALAT1*have been detected in some human cancers [30, 32, 33]. Microphthalmia-associated transcription factor (MITF/MiT) family translocation renal cell carcinoma and other types of tumors, such as perivascular epithelioid cell neoplasm and alveolar soft part sarcoma, are known to harbor *MALAT1* fusion with *TFE3*, *TFEB*, *TFEC*, and *MITF*. *MALAT1* fusions, including *MALAT1::SMG*, *EEF1A1::MALAT1*, and *ASH1L::YY1AP1*, have been detected in nearly 20% of all fusion gene events in gynecologic high-grade neuroendocrine carcinomas [34]. Together with the results presented in this study, these findings suggest that *MALAT1* can be highly available for gene rearrangement to form oncogenic fusions; however, the biological and clinical significance of these *MALAT1* fusions has not been fully investigated.

### Database analysis for the* MALAT1* fusion gene in ChimerSeq

**Fig. 9:**
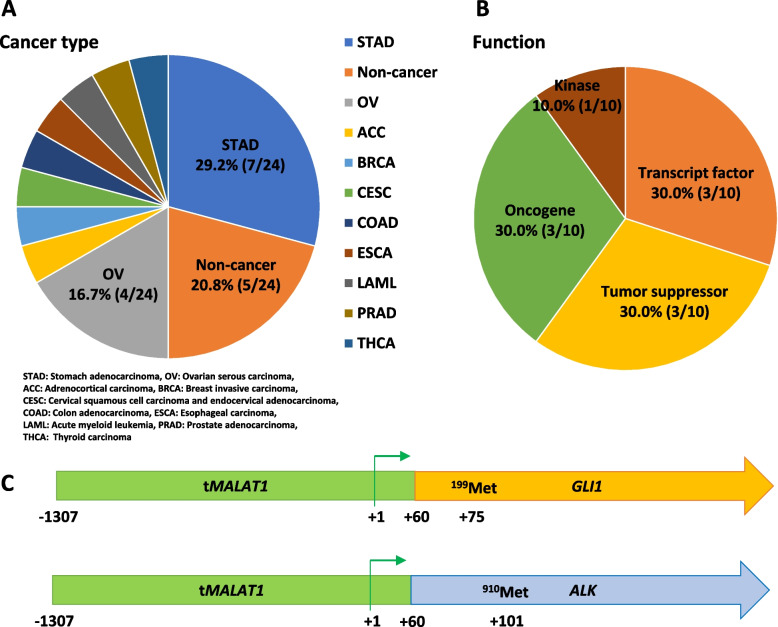
ChimerSeq-based analysis of *MALAT1* fusion genes. **A** A total of 44 entries and 24 unique *MALAT1*/5' fusions are registered in the ChimerSeq database. **B** Fusion gene partners are transcriptional factors, tumor suppressors, oncogenes, and kinases. **C** *MALAT1::ALK* fusion, detected in the thyroid papillary carcinoma, is predicted to have the same breakpoint as that in *MALAT1::GLI1* fusion in the present study. *MALAT1*, metastasis-associated lung adenocarcinoma transcript 1; *GLI1*, glioma-associated oncogene 1

We extracted 44 entries and 24 unique fusions of the *MALAT1*/5'-gene in gastric, ovarian, and other cancers from ChimerDB 4.0, which does not include registration of *MALAT1::GLI1* fusion genes (Fig. 9A). The 3'-fusion gene partners included transcriptional factors, tumor suppressors, oncogenes, and kinases (Fig. 9B). The ChimerSeq system of Chimer DB 4.0 can predict a possible fusion gene structure. Among the 24 fusion genes registered, *MALAT1::ALK* fusion, detected in thyroid papillary carcinoma, showed the same breakpoint as that in *MALAT1::GLI1* fusion (Fig. 9C). The *MALAT1* gene acts as a transcriptional regulatory region when it fuses with a partner gene in a fixed structure. A summary of the 24 fusion genes is provided in Supplementary Table 1.

Comprehensive mRNA sequencing, especially long mRNA sequencing, is a potential tool for the detection of unknown fusion genes [35–37]. Therefore, similar *MALAT1* fusion genes are likely to be discovered in future studies using mRNA sequencing in clinical cancer cases. Moreover, other lncRNA-encoding genes will likely exhibit alternative functions, such as transcriptional regulation, after fusion gene formation. A combinational study with database analysis using ChimerDB 4.0 would therefore enhance the discovery of new fusion gene functions.

## Conclusions

We demonstrated the function of the *MALAT1* gene as a transcriptional regulator. In future clinical studies, oncogenic *MALAT1* fusion genes can be further identified as diagnostic molecular markers through comprehensive mRNA sequencing.

## Supplementary Information

**Additional file 1:** **Supplementary Figure 1.** Pathological findings of plexiform fibromyxoma. A) case of esophageal plexiform fibromyxoma (hematoxylin-eosin staining, 100⨯). B) Tumor cell nuclei are positive for GLI1 (100⨯).

**Additional file 2:** **Supplementary Table 1.** MALAT1 fusion gene prediction by ChimerSeq. **Supplementary Table 2.** Synthesized oligonucleotide used. **Supplementary Table 3.** Primers for plasmid construction. **Supplementary Table 4.** Primers for RT-qPCR. **Supplementary Table 5.** Primers for 5' RACE assay.

**Additional file 3:** Uncropped blots related to Figures.

## Data Availability

All data generated or analyzed during this study are included in this published article and its supplementary information files.

## Abbreviations

5' RACE: Rapid amplification of the 5' cDNA end
CMV: Cytomegalovirus
GLI1: Glioma-associated oncogene 1
HH: Hedgehog
lncRNA: Long non-coding RNA
MALAT1: Metastasis-associated lung adenocarcinoma transcript 1
*MALAT1p*: Original *MALAT1* promoter
MITF/MiT: Microphthalmia-associated transcription factor
ncRNA: Non-coding RNA
NtGLI1: N-terminal truncated GLI1
PFM: Plexiform fibromyxoma
RT-qPCR: Reverse transcription-quantitative PCR
SUFU: Suppressor of fused homolog
*tMALAT1*: *MALAT1* Part of the fusion gene
TSS: Transcriptional start site
