# Supplementary material for: MALAT1 functions as a transcriptional promoter of MALAT1::GLI1 fusion for truncated GLI1 protein expression in cancer
Source: BMC Cancer. 2023 May 10;23:424. doi: 10.1186/s12885-023-10867-6 (PMC10173563; doi:10.1186/s12885-023-10867-6)
Supplement: Supplementary file 1 — Additional file 1: Supplementary Figure 1. Pathological findings of plexiform fibromyxoma. A) case of esophageal plexiform fibromyxoma (hematoxylin-eosin staining, 100⨯). B) Tumor cell nuclei are positive for GLI1 (100⨯). [file 12885_2023_10867_MOESM1_ESM.pptx]

## Slide 1
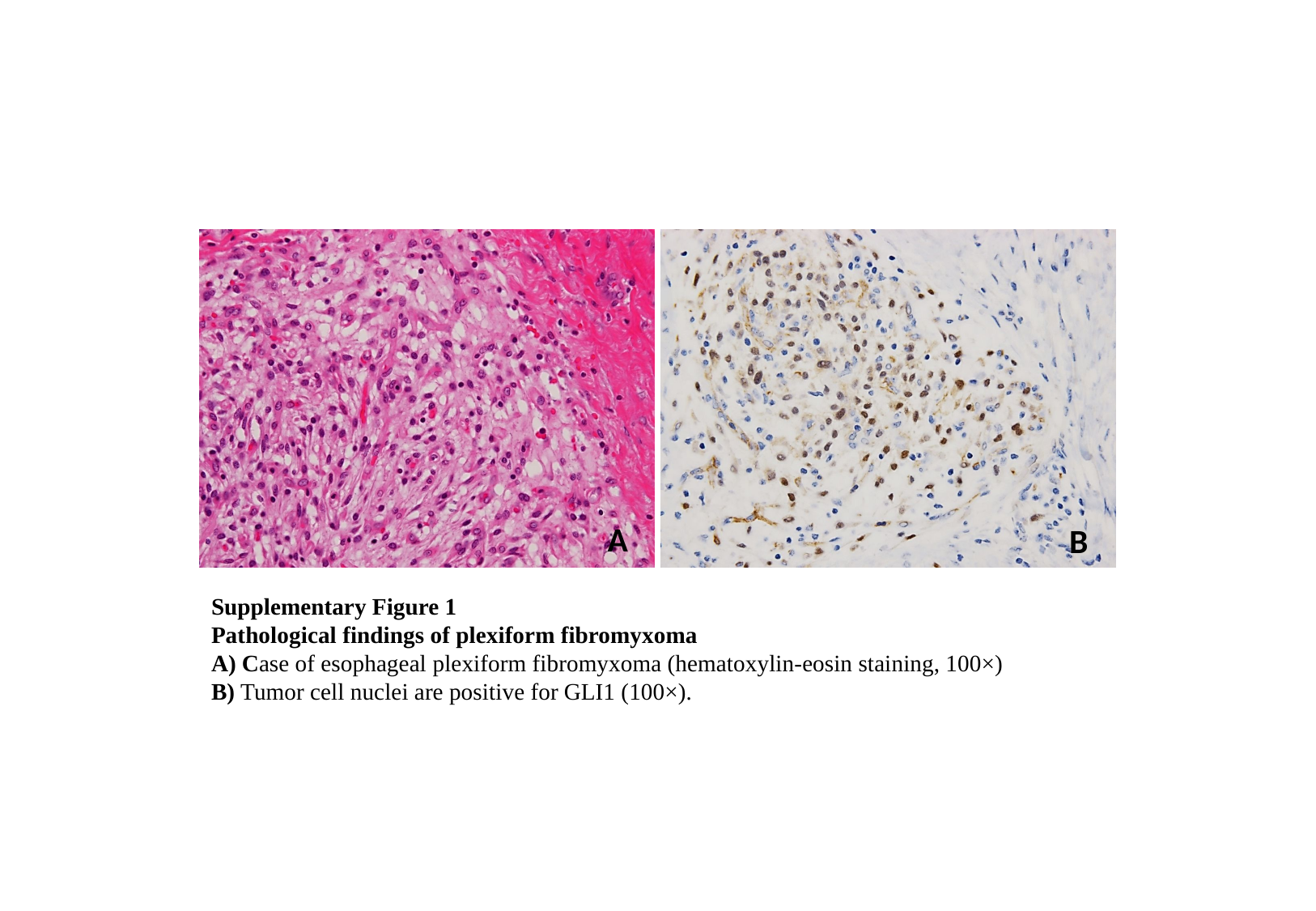

A
B
Supplementary Figure 1
Pathological findings of plexiform fibromyxoma
A) Case of esophageal plexiform fibromyxoma (hematoxylin-eosin staining, 100×)
B) Tumor cell nuclei are positive for GLI1 (100×).
