# Supplementary material for: MALAT1 functions as a transcriptional promoter of MALAT1::GLI1 fusion for truncated GLI1 protein expression in cancer
Source: BMC Cancer. 2023 May 10;23:424. doi: 10.1186/s12885-023-10867-6 (PMC10173563; doi:10.1186/s12885-023-10867-6)
Supplement: Supplementary file 2 — Additional file 2: Supplementary Table 1. MALAT1 fusion gene prediction by ChimerSeq. Supplementary Table 2. Synthesized oligonucleotide used. Supplementary Table 3. Primers for plasmid construction. Supplementary Table 4. Primers for RT-qPCR. Supplementary Table 5. Primers for 5' RACE assay. [file 12885_2023_10867_MOESM2_ESM.docx]

**Supplementary Table 1 MALAT1 fusion gene prediction by ChimerSeq**

| **Fusion pair** | **Gene type** | **Cancer type** | **Barcode ID** | **Gene 5’-Junction** | **Gene 3’-Junction** |
| --- | --- | --- | --- | --- | --- |
| **MALAT1-ALK** | **Oncogene** | **THCA** | **TCGA-DJ-A4UT-01A** | **chr11 : 65269267(+)** | **chr2 : 29451934(-)** |
|  |  |  |  | **chr11 : 65266575(+)** | **chr2 : 29451938(-)** |
|  |  |  |  | **chr11 : 65266581(+)** | **chr2 : 29451932(-)** |
|  |  |  |  | **chr11 : 65269864(+)** | **chr2 : 29451932(-)** |
|  |  |  |  | **chr11 : 65268285(+)** | **chr2 : 29451932(-)** |
|  |  |  |  | **chr11 : 65501796(+)** | **chr2 : 29229068(-)** |
| **MALAT1-CALD1** |  | **STAD** | **TCGA-BR-A4J7-01A** | **chr11 : 65267243(+)** | **chr7 : 134618333(+)** |
|  |  |  | **TCGA-RD-A8N4-01A** | **chr11 : 65267238(+)** | **chr7 : 134618397(+)** |
| **MALAT1-CAPN1** |  | **OV** | **TCGA-23-1107-01A** | **chr11 : 65266585(+)** | **chr11 : 64972154(+)** |
|  |  |  |  | **chr11 : 65266581(+)** | **chr11 : 64972144(+)** |
| **MALAT1-CDC42EP2** |  | **CESC** | **TCGA-EA-A3QE-01A** | **chr11 : 65271158(+)** | **chr11 : 65089886(+)** |
| **MALAT1-CEACAM6** |  | **Non-Cancer** | **TCGA-IP-7968-11A** | **chr11 : 65267143(+)** | **chr19 : 42259438(+)** |
| **MALAT1-CFL1** |  | **STAD** | **TCGA-BR-8690-01A** | **chr11 : 65268285(+)** | **chr11 : 65623713(-)** |
|  |  |  |  | **chr11 : 65266579(+)** | **chr11 : 65623715(-)** |
|  |  |  |  | **chr11 : 65266585(+)** | **chr11 : 65623713(-)** |
|  |  |  |  | **chr11 : 65268249(+)** | **chr11 : 65623713(-)** |
|  |  |  |  | **chr11 : 65268230(+)** | **chr11 : 65623713(-)** |
| **MALAT1-CLU** | **Tumor suppressor gene** | **OV** | **TCGA-13-0720-01A** | **chr11 : 65267154(+)** | **chr8 : 27461914(-)** |
| **MALAT1-DPCR1** |  | **STAD** | **TCGA-R5-A805-01A** | **chr11 : 65266838(+)** | **chr6 : 30917819(+)** |
| **MALAT1-DPF2** |  | **OV** | **TCGA-29-1697-01A** | **chr11 : 65271040(+)** | **chr11 : 65113113(+)** |
|  |  |  |  | **chr11 : 65503569(+)** | **chr11 : 65345642(+)** |
| **MALAT1-DUSP1** | **Tumor suppressor gene** | **Non-Cancer** | **TCGA-BR-6564-11A** | **chr11 : 65267159(+)** | **chr5 : 172198198(-)** |
| **MALAT1-FADS1** |  | **BRCA** | **TCGA-LL-A6FQ-01A** | **chr11 : 65266583(+)** | **chr11 : 61570938(-)** |
|  |  |  |  | **chr11 : 65268908(+)** | **chr11 : 61570931(-)** |
|  |  |  |  | **chr11 : 65268285(+)** | **chr11 : 61570931(-)** |
|  |  |  |  | **chr11 : 65268284(+)** | **chr11 : 61570930(-)** |
|  |  |  |  | **chr11 : 65268907(+)** | **chr11 : 61570930(-)** |
|  |  |  |  | **chr11 : 65268984(+)** | **chr11 : 61571091(-)** |
| **MALAT1-FAM166A** |  | **ACC** | **TCGA-OR-A5JD-01A** | **chr11 : 65269547(+)** | **chr9 : 140139674(-)** |
|  |  |  |  | **chr11 : 65502076(+)** | **chr9 : 137245222(-)** |
| **MALAT1-HAND2** | **Transcription factor** | **Non-Cancer** | **TCGA-FL-A1YM-11A** | **chr11 : 65267968(+)** | **chr4 : 174448210(-)** |
| **MALAT1-IGF2** | **Oncogene** | **OV** | **TCGA-04-1338-01A** | **chr11 : 65266546(+)** | **chr11 : 2152009(-)** |
| **MALAT1-IGHG1** |  | **STAD** | **TCGA-BR-A4PD-01A** | **chr11 : 65267284(+)** | **chr14 : 106208511(-)** |
| **MALAT1-MYH11** | **Oncogene** | **Non-Cancer** | **TCGA-HU-A4GH-11A** | **chr11 : 65268545(+)** | **chr16 : 15815340(-)** |
| **MALAT1-PCNP** |  | **STAD** | **TCGA-HU-A4GN-01A** | **chr11 : 65267149(+)** | **chr3 : 101293065(+)** |
| **MALAT1-PPP1R1B** | **Tumor suppressor gene** | **ESCA** | **TCGA-L5-A8NR** | **chr11 : 65267145(+)** | **chr17 : 37783255(+)** |
| **MALAT1-RPL13A** |  | **LAML** | **TCGA-AB-2986_700GJAAXX_1** | **chr11 : 65267237(+)** | **chr19 : 49994993(+)** |
| **MALAT1-RPS12** |  | **COAD** | **TCGA-CM-4748-01A** | **chr11 : 65267132(+)** | **chr6 : 133138110(+)** |
| **MALAT1-SCO2** |  | **STAD** | **TCGA-BR-A4PD-01A** | **chr11 : 65267159(+)** | **chr22 : 50964742(-)** |
| **MALAT1-SPZ1** | **Transcription factor** | **PRAD** | **TCGA-V1-A9OT** | **chr11 : 65268285(+)** | **chr5 : 79615956(+)** |
|  |  |  |  | **chr11 : 65272490(+)** | **chr5 : 79615956(+)** |
|  |  |  | **TCGA-V1-A9OT-01A** | **chr11 : 65500814(+)** | **chr5 : 80320137(+)** |
| **MALAT1-TFEB** | **Transcription factor** | **renal cell carcinoma** | **AJ535462** | **chr11 : 65266903()** | **chr6 : 41659049()** |
| **MALAT1-TYMP** |  | **STAD** | **TCGA-BR-A4PD-01A** | **chr11 : 65267159(+)** | **chr22 : 50964742(-)** |

**STAD: Stomach adenocarcinoma, OV: Ovarian serous carcinoma, ACC: Adrenocortical carcinoma, BRCA: Breast invasive carcinoma, CESC: Cervical squamous cell carcinoma and endocervical adenocarcinoma, COAD: Colon adenocarcinoma, ESCA: Esophageal carcinoma, LAML: Acute myeloid leukemia, PRAD: Prostate adenocarcinoma, THCA: Thyroid carcinoma**

**Supplementary Table 2 Synthesized oligonucleotide used**

|  | Sequence (5’ – 3’) |
| --- | --- |
| MALAT_513 | aaaaaAAGCTTctgtcttgggagcaagtcgcaggactgcaagcagttgggggagaaagtccgccattttgccacttctcaaccgtccctgcaaggctggggctcagttgcgtaatggaaagtaaagccctgaactatcacactttaatcttccttcaaaaggtggtaaactatacctactgtccctcaagagaacacaagaagtgctttaagaggtattttaaaagttccgggggttttgtgaggtgtttgatgacccgtttaaaatatgatttccatgtttcttttgtctaaagtttgcagctcaaatctttccacacgctagtaatttaagtatttctgcatgtgtagtttgcattcaagttccataagctgttaagaaaaatctagaaaagtaaaactagaacctatttttaaccgaagaactactttttgcctccctcacaaaggcggcggaaggtgatcGAATTCaaaaa |
| MALAT_332 | aaaaaAAGCTTaacacaagaagtgctttaagaggtattttaaaagttccgggggttttgtgaggtgtttgatgacccgtttaaaatatgatttccatgtttcttttgtctaaagtttgcagctcaaatctttccacacgctagtaatttaagtatttctgcatgtgtagtttgcattcaagttccataagctgttaagaaaaatctagaaaagtaaaactagaacctatttttaaccgaagaactactttttgcctccctcacaaaggcggcggaaggtgatcGAATTCaaaaa |
| MALAT_171 | aaaaaAAGCTTgcattcaagttccataagctgttaagaaaaatctagaaaagtaaaactagaacctatttttaaccgaagaactactttttgcctccctcacaaaggcggcggaaggtgatcGAATTCaaaaa |

**Supplementary Table 3 Primers for plasmid construction**

|  | **Primer sequence (5’ – 3’)** | |
| --- | --- | --- |
| **Truncated MALAT1 - GLI1 expression plasmids** | |  |
| MALfus | ATATACGCGTTGACAcgcagcctgcagcccga | ttccaagggttcctcaagctccgcctgcc |
| MALfus_CMV | GTTTAAACTTAAGCTcgcagcctgcagcccga | ttccaagggttcctcaagctccgcctgcc |
| GLIfus | TGTCAACGCGTATATCTGGCCCGTACA | gaggaacccttggaaggtgatatgtcca |
| GLIfus_CMV | AGCTTAAGTTTAAACGCTAGCCAGCTTGGG | gaggaacccttggaaggtgatatgtcca |
| GLIFL_CMV | cacaAAGCTTatgttcaactcgatgaccccacc | caGGTAAGCTTAAGTTTAAACGCTAGCCAGCTT |
| GLI129_CMV | aaaaAAGCTTggcaccatgagcccatctct | caGGTAAGCTTAAGTTTAAACGCTAGCCAGCTT |
| GLI199_CMV | acaAAGCTTgaggaacccttggaaggtgatatgtc | caGGTAAGCTTAAGTTTAAACGCTAGCCAGCTT |
| GLI199 | aaaACGCGTgaggaacccttggaaggtgatatgtc | caGGTAAGCTTAAGTTTAAACGCTAGCCAGCTT |
| GLI_K180R_F | caacttgccagctgaGgtctgagctggacat | atgtccagctcagacCtcagctggcaagttg |
| MALfus_168 | ATATACGCGTTGACAcgcagcctgcagcccga | ttccaagggttcctcttacgttaaaaacttaacgc |
| MALfus_-56 | ATATACGCGTTGACAcgcagcctgcagcccga | ttccaagggttcctcattcgatcaccttccgccgc |
| -828/+13_F | ATATACGCGTTGACAccatcctaacctatacagcgtcac | cccagtcctttacagaagtctcg |
| MALpfus | ctgtaaaggactggggccccgc | TGTCAACGCGTATATCTGGCCCGTACA |
| **pGL4 reporter plasmids** | |  |
| pGL4MALAT | tcggcggccaagcttcgcagcctgcagcccga | tttggcatcttccatatcaccttccaagggttcctcaagc |
| FOXL1mt_F | tgttctccgtctataTTtacgcctcgcccgag | ctcgggcgaggcgtaAAtatagacggagaaca |
| SP5mt_F | gaactactttttgccAAcctcacaaaggcggc | gccgcctttgtgaggTTggcaaaaagtagttc |
| ZBTBmt_F | gaaaagtaaaactagTCcctatttttaaccga | tcggttaaaaataggGActagttttacttttc |
| TATAmt_F | agttgttctccgtctCGGaatacgcctcgcccg | cgggcgaggcgtattCCGagacggagaacaact |
| pGL4MALAT_pF2 | tcggcggccaagcttccatcctaacctatacagcgtcactaatctc |  |
| **SUFU expression plasmid** | |  |
| SUFU | aaaaaGCGGCCGCGatggcggagctgcggcctag | aaaAGATCTctagtgtagcggactgtcgaacacc |
| **myc-tagged GLI1 expression plasmid** | |  |
| myc-GLI-FL_F | CTGAAGCCCGGGCGGatgttcaactcgatgacccc | TCCTGCAGCCCGGGGCTGTGATGGATGAGATTCCC |
| myc-GLI199_F | CTGAAGCCCGGGCGGgaggaacccttggaaggtga | TCCTGCAGCCCGGGGCTGTGATGGATGAGATTCCC |
| **Sequencing primer** | |  |
| MALp_seqF | AATTGCATGAAGAATCTGCTTAGGG |  |
| MALp_seqR | ACAGCTAAGATAGCAGCACAACTC |  |

**Supplementary Table 4 Primers for RT-qPCR**

| **Target genes** | **Primer sequence (5’ – 3’)** | | **References** |
| --- | --- | --- | --- |
| **CCND1** | TGAAGGAGACCATCCCCCTG | TGTTCAATGAAATCGTGCGG | 1 |
| **MYC** | TGAGGAGACACCGCCCAC | CAACATCGATTTCTTCCTCATCTTC | 2 |
| **GLI1 (exons1-2)** | AGACTCCAGCCCTGGACCGC | GGCGTCTCAGAGGAGGGTGTG | 3 |
| **PTCH1** | AATGGGTCCACGACAAAGCCGACTA | TCCCGCAAGCCGTTGAGGTAGAAAG | 4 |
| **SOSTDC1** | TCGGAACACTCGGGTTCAAGTGG | CAGCACACACCAGCTCCTTCAGA | 4 |
| **FOXS1** | CTGGTGAGCCAAGGACAACCACA | AAATCCCAAGAGGCCCTGCTTCC | 4 |
| **HHIP1** | TTTTACACTTGCCGAGGCCATATTCCA | AGCACAACCCACCATCTTTTCTTGCAT | 4 |
| **HPRT** | AATTATGGACAGGACTGAACGTC | CGTGGGGTCCTTTTCACCAGCAAG | 5 |

**References**

1. Amatori S, Persico G, Fanelli M. Real-time quantitative PCR array to study drug-induced changes of gene expression in tumor cell lines. J Cancer Metastasis Treat 2017;3:90-9. doi: 10.20517/2394-4722.2017.22.
2. Kumar N, Basundra R, Maiti S. Elevated polyamines induce c-MYC overexpression by perturbing quadruplex-WC duplex equilibrium. Nucleic Acids Res. 2009;37:3321-3331. doi: 10.1093/nar/gkp196.
3. Wang XQ, Rothnagel JA. Post-transcriptional regulation of the gli1 oncogene by the expression of alternative 5' untranslated regions. J. Biol. Chem. 2001;276:1311-1316. doi: 10.1074/jbc.M005191200.
4. Diao Y, et al. Identification of novel GLI1 target genes and regulatory circuits in human cancer cells. Mol. Oncol. 2018;12:1718-1734. doi: 10.1002/1878-0261.12366.
5. Suzuki H, et al. Paracrine upregulation of VEGF receptor mRNA in endothelial cells by hypoxia-exposed hep G2 cells. Am. J. Physiol. 1999;276:G92-G97. doi: 10.1152/ajpgi.1999.276.1.G92.

**Supplementary Table 5 Primers for 5' RACE assay**

|  | **Primer sequence (5’ – 3’)** |
| --- | --- |
| GLI_RACE_R2 | gattacgccaagcttCTGCATTGCCAGTCATTTCCACACC |
| LUC_RACE_R | TAGGTAATGTCCACCTCGATATGTG |
