# Supplementary material for: MALAT1 functions as a transcriptional promoter of MALAT1::GLI1 fusion for truncated GLI1 protein expression in cancer
Source: BMC Cancer. 2023 May 10;23:424. doi: 10.1186/s12885-023-10867-6 (PMC10173563; doi:10.1186/s12885-023-10867-6)
Supplement: Supplementary file 3 — Additional file 3: Uncropped blots related to Figures. [file 12885_2023_10867_MOESM3_ESM.pptx]

## Slide 1
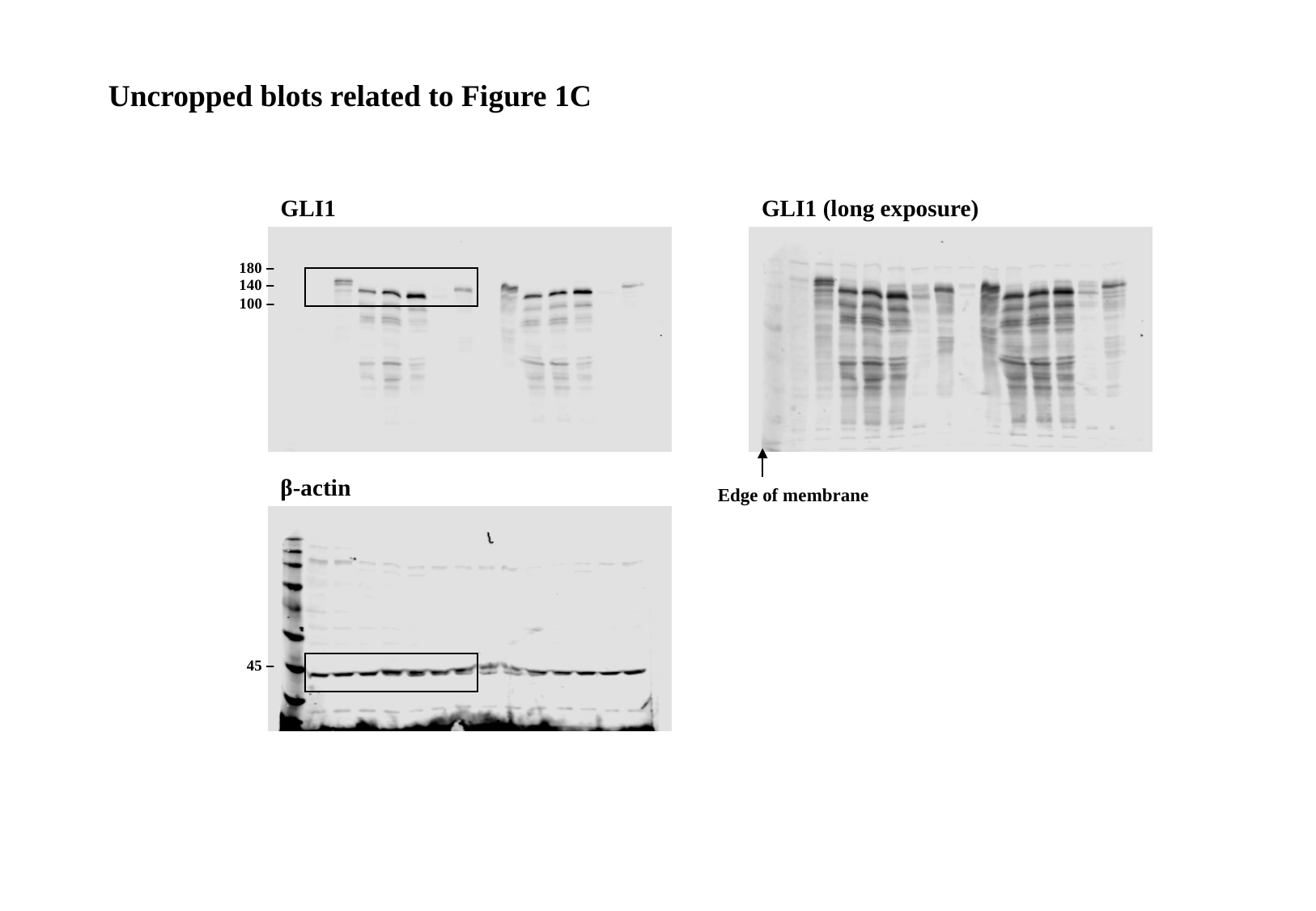

Uncropped blots related to Figure 1C
GLI1 (long exposure)
GLI1
180 –
140 –
100 –
β-actin
Edge of membrane
45 –

## Slide 2
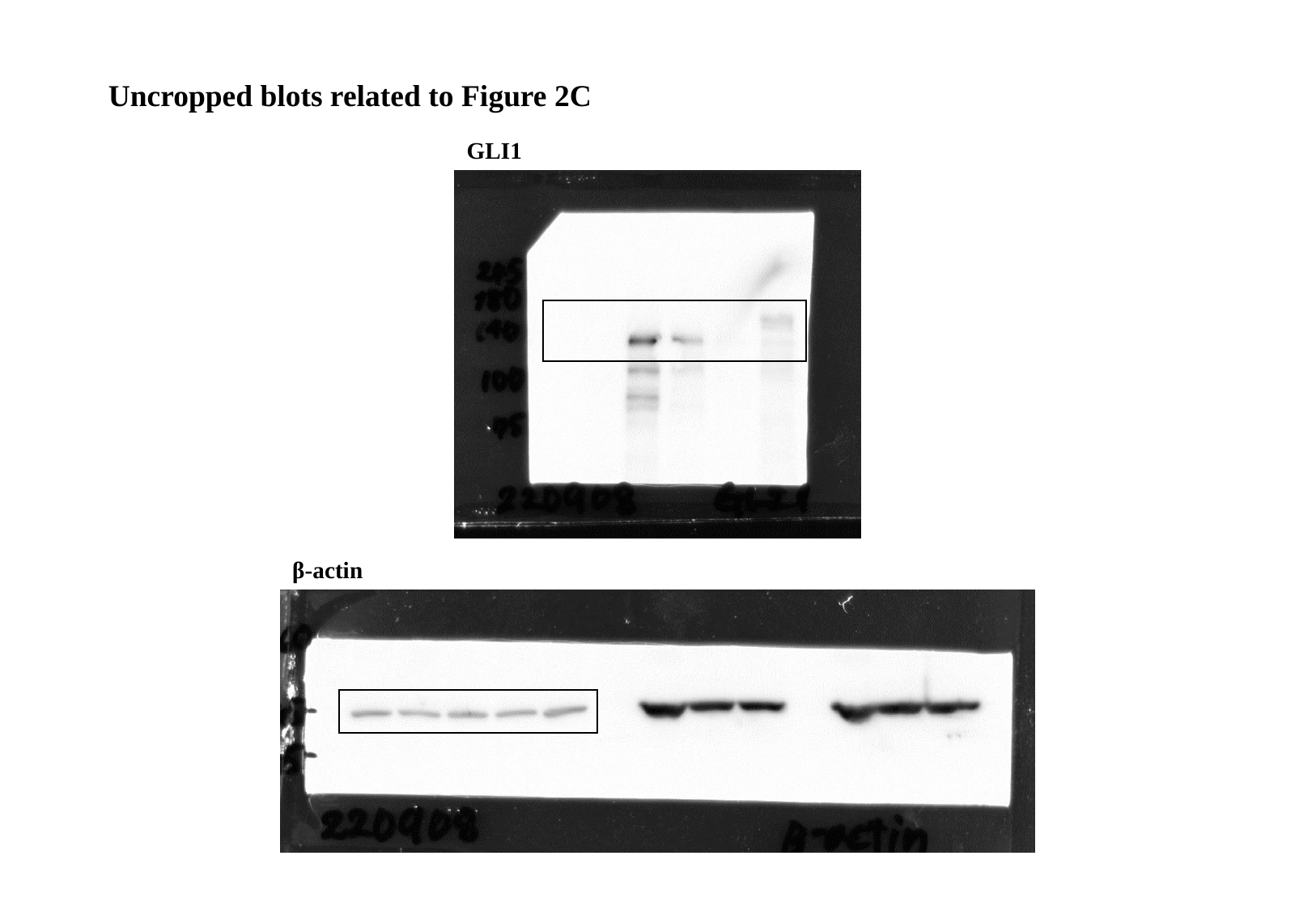

Uncropped blots related to Figure 2C
GLI1
β-actin

## Slide 3
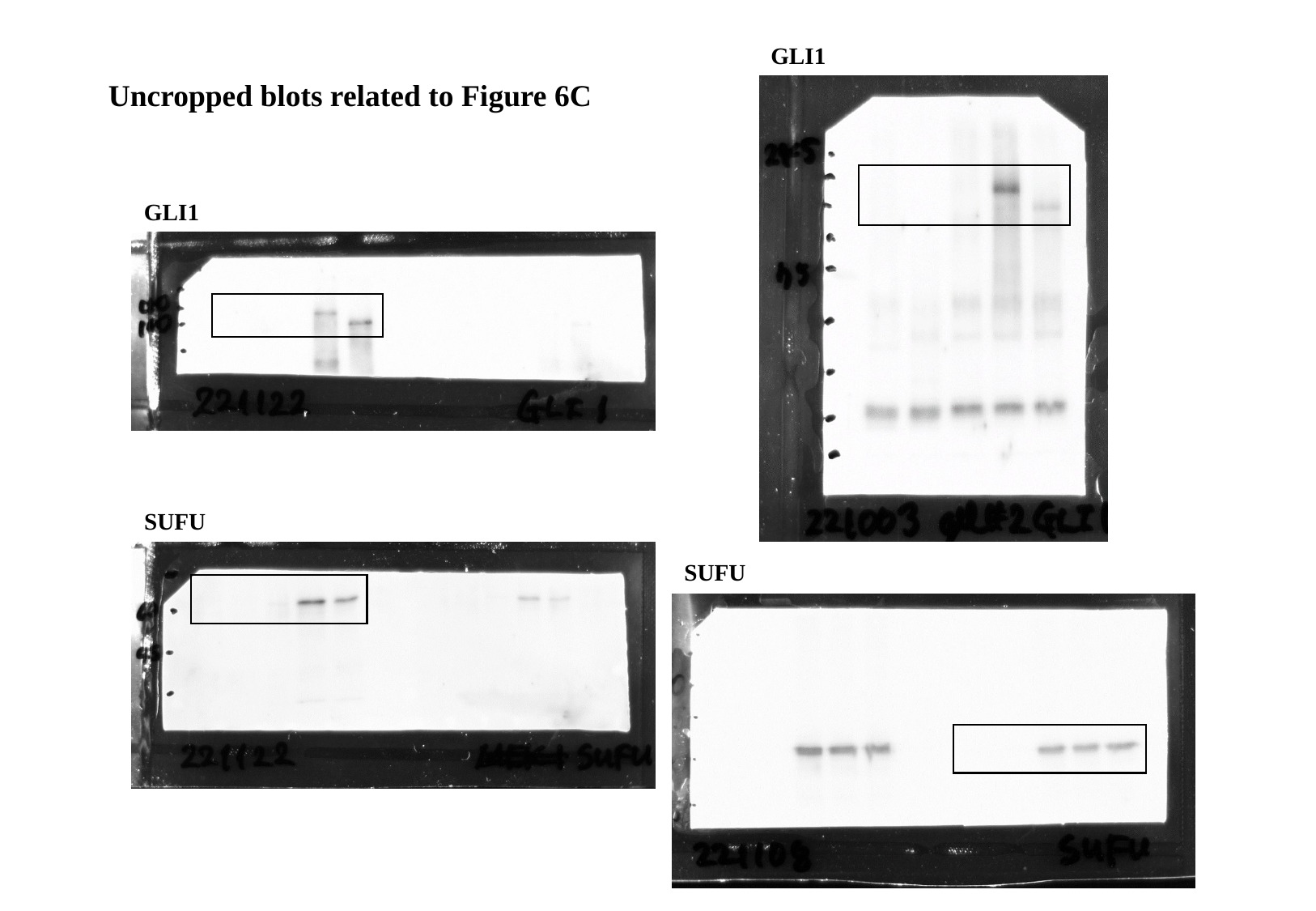

GLI1
Uncropped blots related to Figure 6C
GLI1
SUFU
SUFU

## Slide 4
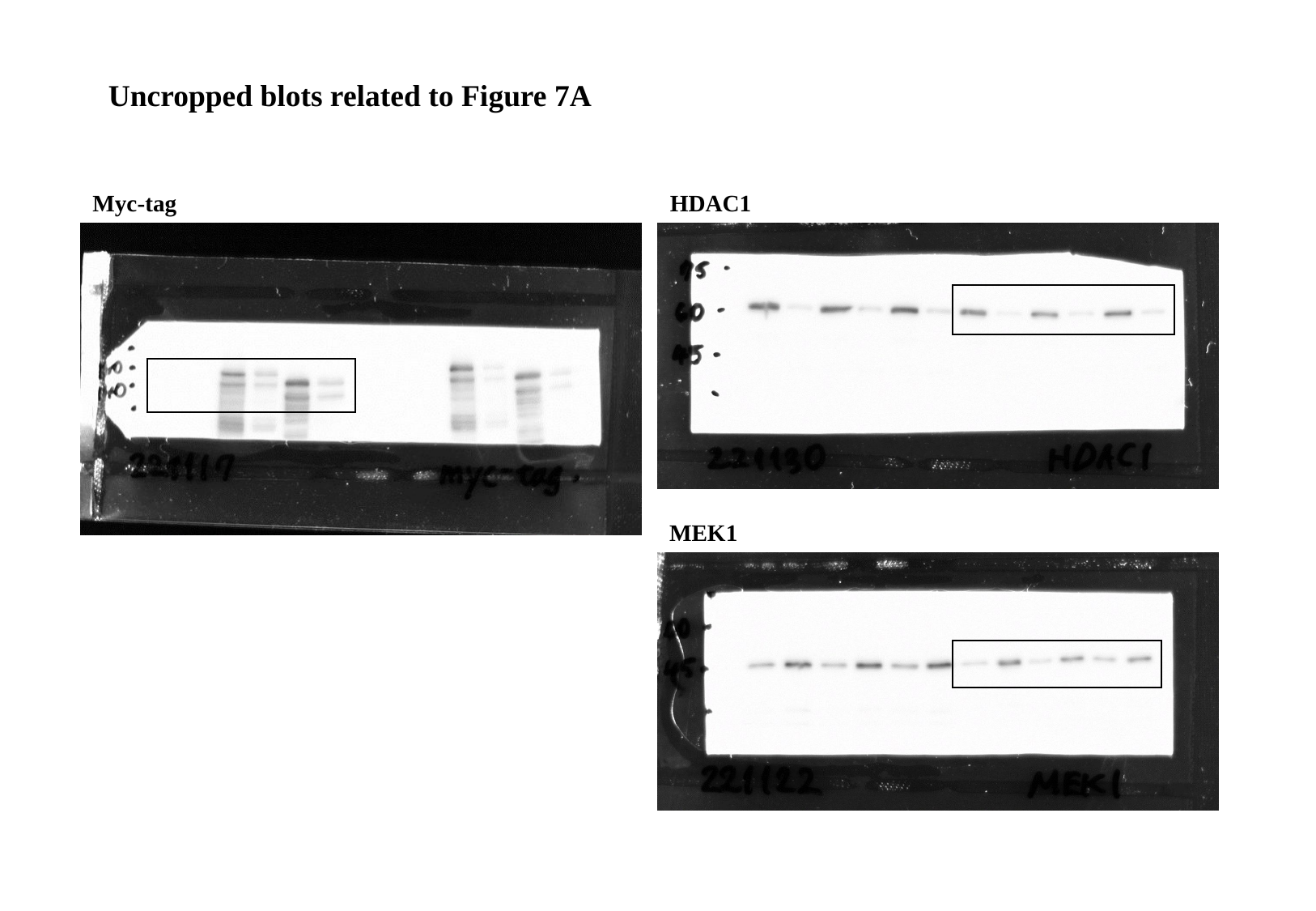

Uncropped blots related to Figure 7A
Myc-tag
HDAC1
MEK1
